# Interleukin-6 (-174G/C), Interleukin-1β (-511 C/T), and Apolipoprotein B-100 (2488 C/T) Gene Polymorphism in Pre-Eclampsia

**DOI:** 10.3390/medicina60081307

**Published:** 2024-08-13

**Authors:** Muhammad Naveed Najeeb, Umaira Munir, Muhammad Ameer Hamza, Sadia Mehmood, Javed Anver Qureshi, Tahir Maqbool

**Affiliations:** 1 Institute of Molecular Biology and Biotechnology, The University of Lahore, Lahore 40100, Punjab, Pakistan; dr.naveednajeeb@gmail.com; 2 Quaid-e-Azam Medical College, Bahawalpur 63100, Punjab, Pakistan; drumairanaveed@gmail.com; 3 Shahida Islam Medical College, Lodhran 59320, Punjab, Pakistan; ameerhamxa902@gmail.com; 4 Bakhtawar Ameen Medical College, Multan 60800, Punjab, Pakistan; saadiah786@yahoo.com

**Keywords:** pre-eclampsia, gestational hypertension, proteinuria, restriction fragment length polymorphism, Hardy–Weinberg equilibrium

## Abstract

*Background and objectives*: Pre-eclampsia (PE) is a pregnancy-specific condition characterized by significant health risks for pregnant women worldwide due to its status as a multi-organ disorder. High blood pressure (hypertension) with or without proteinuria is usually considered an initial clinical sign of PE. The pathogenesis of pre-eclampsia is highly complex and likely involves multiple factors, including poorly developed uterine spiral arterioles, immunological issues, placental ischemia or infarction, and genetic abnormalities. Inflammatory cytokine production, regulated by cytokine gene polymorphisms, is one of the factors likely contributing to the development of PE. The present study aimed to assess IL-6, IL-1β, and Apo B-100 gene polymorphism and to evaluate the association of these polymorphisms with PE. *Materials and Methods*: This cross-sectional observational study involved 99 participants aged 16 to 45 years from Bahawal Victoria Hospital Bahawalpur, Punjab, Pakistan. The participants were divided into three groups: Group 1 (PE with severe hypertension), Group 2 (PE with hypertension), and Group 3 (control), each comprising 33 individuals. Maternal blood samples were collected, DNA was extracted, and molecular genetic analysis of the IL-6, IL-1β, and Apo B-100 genes was performed using the PCR-RFLP method. Allelic frequencies were compared, and statistical analysis was conducted using SPSS 25, applying the Hardy–Weinberg equation and chi-square test to evaluate the results. *Results*: There are differences in the distribution of allelic frequencies for IL-6 -174G/C (CC, GC, GG), IL-1β-511C/T (CC, CT, TT), and Apo B-100 2488 C/T (CC, CT, TT) between pre-eclamptic patients and the control group. The analysis using the Hardy–Weinberg equilibrium and chi-square test showed an association between the IL-6-174 G/C polymorphism and the severity of pre-eclampsia. *Conclusions*: The polymorphisms of the IL-6, IL-1β, and Apo B-100 genes revealed different alleles. The IL-6 gene alone was found to be in disequilibrium according to the Hardy–Weinberg equation, indicating a potential link to the severity of pre-eclampsia in the population studied.

## 1. Introduction

Pre-eclampsia is a prevalent pregnancy complication worldwide, contributing significantly to maternal and fetal morbidity and mortality [1,2,3]. This disorder is marked by vascular endothelial dysfunction and vasospasm, typically arising after 20 weeks of gestation and sometimes appearing postpartum [2,4,5]. Diagnosis involves elevated blood pressure readings (>140/90) taken six hours apart with or without significant proteinuria (>300 mg/24 h) [1,2,6]. Globally, pre-eclampsia incidence ranges from 2 to 10% of pregnancies, with higher rates in developing countries (1.8–16.7%) compared to developed nations (0.4%) [2]. Approximately 10% of pregnant women without prior hypertension develop pre-eclampsia post-20 weeks, with a 5.4% prevalence in those with chronic hypertension [2,4]. In severe cases, the mother may develop comorbidities such as hepatic alterations (HELLP syndrome), edema, disseminated vascular coagulation (DIC), and eclampsia, particularly targeting the brain (cerebral edema) [2,3,4,5,6]. For the fetus, the main complications associated with PE include growth restriction, leading to low birth weight, prematurity, and fetal death [2,3,5,6].

Though the pathophysiology of pre-eclampsia has been better understood, the fundamental mechanism, which is probably multifactorial, remains unexplained [3,6]. During a normal pregnancy, cytotrophoblasts infiltrate the uterine myometrium, creating a dense network of vascular anastomoses that eventually supply blood to the fetus and placenta [7,8]. However, cytotrophoblasts in pre-eclamptic patients do not develop the invasive phenotype required to form these strong anastomoses, which leads to a reduced and shallow endovascular invasion of the spiral arteries [3,6]. The tiny calibre of these aberrant blood arteries and the loss of elasticity result in placental ischemia and inadequate oxygen transport. A combination of abnormal placentation and ischemia releases pro-inflammatory proteins (cytokines like interleukins) into the maternal circulation, resulting in endothelial dysfunction and the clinical symptoms observed in pre-eclampsia patients [6,8]. Placental ischemia brought on by uteroplacental underperfusion also increases the production of antiangiogenic factors such as soluble endoglin (sEng) and soluble fms-like tyrosine kinase 1 (sFlt-1). By acting as scavengers and lowering circulating angiogenic factors like PIGF and VEGF levels, these substances negatively impact endothelial function and the angiogenic balance. This imbalance results in the clinical manifestation of the disease [3,6,8,9].

Feto-maternal immunity has become one of the most researched topics. Research points to a potential correlation between pro- and anti-inflammatory factor imbalance and the development of pre-eclampsia, which triggers a systemic response involving vascular endothelium [3,6]. Immune cells that assist spiral artery remodelling and fetal trophoblast growth during a healthy pregnancy include regulatory T lymphocytes, dendritic cells, and natural killer cells in the decidua. These cells also help maintain immunotolerance [7,10]. The mother’s body is able to better tolerate the fetal transplant thanks to this local immunological milieu [7,10,11].

Unlike normal pregnancy, which is characterized by immunosuppression, pre-eclamptic pregnancy is characterized by increased immunological activation [11,12]. Via cytokine activity, Th1 cells, NK cells, and self-reactive B cells induce an inflammatory response that impairs spiral artery remodelling and trophoblast invasion. Reduced trophoblast invasion and transient ischemia followed by reperfusion both increase oxidative stress, which damages endothelium and triggers inflammation [6,8,11]. By stimulating endothelial cells and other cell types, producing pro-inflammatory cytokines, and releasing cellular debris from the syncytiotrophoblast (STB), this pathological state causes a systemic inflammatory response [8,10]. Simultaneously, inflammation is driven by pro-inflammatory cytokines such as IL-1β, TNF-α, IL-6, IL-8, and IL-18, primarily secreted by maternal immune cells [11]. Additionally, the trophoblast contributes to inflammation by producing interleukins (IL-1β, IL-2, IL-4, IL-6, IL-8, IL-12, TGFβ1, and TNF-α), chemokines (MCP-1), and adhesion molecules (ICAM-1 and VCAM-1) [8,9,11].

Recently, it was discovered that the decidua’s pro- and anti-inflammatory macrophage balance must be maintained for a pregnancy to proceed normally [7,8]. Pre-eclampsia patients may experience disrupted placentation due to the disruption of tissue macrophages in the endometrium caused by an increase in nonclassical macrophage subpopulations [9]. Pre-eclampsia is characterized by unregulated M1 macrophage activity and the inhibition of the natural transition towards M2 macrophages during the second trimester. This results in a reduction in IL-4 and IL-10 levels and an increase in pro-inflammatory cytokine production [9,12].

It is also new information that the inflammatory response associated with placental failure in pre-eclampsia is mediated by cytosolic multiprotein complexes called inflammasomes, which are expressed in placental cells [13,14]. Studies have revealed that peripheral monocytes from pre-eclamptic women express higher levels of inflammasomes, such as NLRP1 and NLRP3. The NLRP3 inflammasome in syncytiotrophoblasts can be triggered by elevated levels of total cholesterol and uric acid, which are host-derived damage-associated molecular patterns (DAMPs) [9,14,15]. In several observational studies, uric acid, lipids, and several minerals (calcium and magnesium) are associated with the development of PE, whereby hyperuricemia, dyslipidemias, and deranged minerals are also linked to ROS and inflammation [3,16,17]. The most extensively researched inflammasome, NLRP3, contributes to the inflammatory milieu in pre-eclampsia by releasing IL-1β and IL-18. It has been suggested that IL-6 is involved in NLRP3 inflammasome activation and subsequent IL-1β production from innate immune cells and has a crucial role in synovial inflammation [9,13,14].

Another factor that is involved in the pathophysiology of pre-eclampsia is dyslipidemia [16,18]. During pregnancy, complex changes in lipid metabolism likely contribute to maternal dyslipidemia [18,19]. The pre-eclamptic patients show elevated lipid biomarkers [16,19]. Cholesterol is a key component of lipids and plasma lipoproteins, which transport lipids [19]. The hydrophobic core of lipoproteins is made up of esterified cholesterol and triacylglycerols while the hydrophilic surface layer consists of unesterified cholesterol, phospholipids, and Apolipoproteins [20]. The lipoproteins are chylomicrons, very-low-density lipoprotein (VLDL), intermediate-density lipoprotein (IDL), low-density lipoprotein (LDL), and high-density lipoprotein (HDL) [21]. Apo B is a component of all atherogenic or potentially atherogenic particles, including VLDL, IDL, LDL, and lipoprotein(a) [Lp(a)], with each particle containing one molecule of apo B [20,21]. This makes apo B a direct measure of the number of atherogenic lipoprotein particles in circulation. Apo B-containing lipoproteins play a crucial role in atherogenesis, promoting plaque formation within arteries [18,20]. Recent studies have shown that atherogenesis and plaque formation are involved in the pathogenesis of pre-eclampsia [18,19]. Measuring apo B provides a direct indicator of the number of circulating atherogenic particles, as each hepatic-derived lipoprotein particle contains one apo B molecule. Additionally, some studies have found that measuring apo B improves pre-eclampsia prediction in patients [20].

Genetic polymorphisms are under intense scrutiny as they affect pathways like angiogenesis, inflammation, lipid metabolism, and vascular function [22]. Elevated cytokine levels, particularly IL-1β, IL-2, IL-6, and IFN-γ, in pre-eclampsia exacerbate harmful Th1 immunity, thereby damaging maternal endothelium and hindering trophoblast invasion [23]. IL-6, produced by various cells, plays a significant role in PE, with elevated levels contributing to systemic inflammation. IL-6 cytokine is synthesized through the IL-6 gene. Increased IL-6 expression in pre-eclampsia may exacerbate endothelial dysfunction and influence decidual macrophages involved in trophoblast invasion [24]. IL-1β also contributes to PE pathophysiology, stimulating the production of other cytokines and amplifying the inflammatory response. Genetic variations in IL-1β may impact the inflammatory environment, influencing PE development and severity [22]. ApoB-100 gene variations are linked to dyslipidemia and endothelial dysfunction, integral to PE pathophysiology. Mutations in ApoB-100 can lead to hypocholesterolemia or hypercholesterolemia, affecting lipid transport and cholesterol levels [25]. Research on genetic links to pre-eclampsia has identified various gene polymorphisms and molecular markers, highlighting the multifaceted nature of this disease.

To assist in the diagnosis, treatment, and prevention of pre-eclampsia, the study set out to investigate some of the genetic markers involved in the pathophysiology of this disorder. Specifically, the interleukin-6 (IL-6)-174 G/C polymorphism, interleukin-1β (IL-1β) gene-511C/T polymorphism, and Apolipoprotein B-100 (Apo B-100) 2488 C/T polymorphisms in pre-eclamptic and healthy pregnant women were examined. The findings of this study aim to enhance the understanding of the genetic factors associated with pre-eclampsia, a critical and emerging research topic worldwide.

## 2. Material and Methods

This cross-sectional study conducted from June 2023 to February 2024 included 99 women aged 20 to 45 with singleton pregnancies who developed hypertension and proteinuria during pregnancy, while excluding those with chronic hypertension, gestational diabetes, cardiovascular disorders, renal disease, immunological disorders, PCOS, metabolic disorders, multiple pregnancies, active labour, early rupture of membranes, or febrile illnesses. Ethical approval was obtained from the Ethical Board of Quaid-e-Azam Medical College, Bahawalpur. Written and informed consent was acquired from all participants, and a detailed questionnaire was used to collect demographic and clinical information. The sampling method was non-probability convenience sampling. According to NICE guidelines, the patients were divided into three groups based on blood pressure measurements—Group 1, PE with severe hypertension (i.e., BP ≥ 160/110); Group 2, PE with hypertension (i.e., BP ≥ 140/90); and Group 3, normal (i.e., BP ≤ 120/80) [26]. Data collection involved clinical examinations by medical professionals at Bahawal Victoria Hospital, Bahawalpur. Blood samples were collected under aseptic conditions, with 5 mL drawn from the median cubital vein and stored in EDTA tubes for immune genetic analysis. High-purity analytical laboratory chemicals, molecular biology-grade reagents, and diagnostic kits were arranged.

### 2.1. DNA Extraction

Chromosomal DNA was extracted and purified from the blood samples of the study subjects using commercial ISO 13485 2016 certified “WizPrep gDNA Mini Kit” cat no. W71050-100 from Wizbiosolutions, Seongnam-si, Republic of Korea [27]. By using silica membrane technology, the gDNA Mini Kit (Blood) does not require the laborious procedures that are usually involved with loose resins or slurries. The purified DNA obtained from this kit is suitable for PCR and restriction endonuclease digestion. The kit method was used as per vendor instructions. The method is based on 5 steps involving the lysis of whole blood using proteinase K and heating the sample for 10 min at 56 °C, followed by a binding step, washing, and elution of purified DNA.

### 2.2. DNA Quantification

The Nanodrop method was utilized to ascertain the concentration of pure DNA. In some instances, the concentration of purified DNA at a 260 nm wavelength was determined using the UV absorbance spectrometry technique [28].

### 2.3. DNA Amplification by Polymerase Chain Reaction (PCR)

The DNA fragments of genes (IL-6, IL-1β, and Apo B-100) were amplified using Polymerase chain reaction (PCR) [29] and specific primers. The primers are listed in Table 1.

A 100 IU tube with a 50 IU reaction volume was used to conduct the polymerase chain reaction. The reaction mixture contained 2.5 IU Taq DNA polymerase, 1 M betaine, 2 mM magnesium chloride, 200 uM of the dNTP, and reaction buffer. Each PCR mixture contained 0.8 microlitres of each primer, 8 microlitres of the master mix, and 5 microlitres of distilled water. Adding 100 ng, or nearly 2 microlitres, of DNA was necessary to start the PCR.

PCR amplification conditions were as follows:

The melting temperature (Tm) for 10 min was 95 °C.

This was followed by 35 cycles of the following:

30 s at 95 °C;

30 s at 54 °C;

30 s at 72 °C.

Finally, there was a final extension for 7 min at 72 °C.

### 2.4. Restriction Fragment Length Polymorphism (RFLP)

The amplified DNA (amplicon) of a specific gene product was digested with specific restriction enzymes. IL6, IL-1β, and Apo B-100 gene products (198 bp, 304 bp, and 710 bp). were restricted separately with SfaNI, AvaI, and XbaI restriction enzymes, respectively. The entire Restriction Fragment Length Polymorphism (RFLP) procedure adhered to the protocol outlined by Pacheco-Romero et al. (2020) [30].

### 2.5. Agarose Gel Electrophoresis

Electrophoretic analysis of extracted genomic DNA, PCR amplified products, and restricted amplicons were separated on agarose gel [30]. The electrophoresis was conducted in 1x buffer for 30–40 min at 100 volts. The resolved PCR products were then visualized on the gel using a UV transilluminator.

One gramme of agarose was microwaved for two to five minutes in 100 millilitres of TBE buffer (0.89 M Tris-Borate, 0.025 M EDTA) to prepare the agarose gel. After that, ethidium bromide was added to the gel to help with DNA visualization.

### 2.6. Statistical Analysis

The analysis of the data was performed with SPSS 26.0. The information was presented as means ± standard deviation (SD) or, if appropriate, as frequency and percentages. The three groups were subjected to a two-way analysis of variance (ANOVA) [31].

In genetic investigations, Hardy–Weinberg equilibrium was used. To determine if the population under study is in HWE, a chi-square (χ^2^) test was used to compare the expected genotype frequencies under HWE to the observed frequencies [32]. *p* < 0.05 was regarded as statistically significant.

### 2.7. Hardy–Weinberg Equilibrium (HWE) Analysis

To analyze whether the genotype distributions for the given genes in different groups were in Hardy–Weinberg equilibrium, we followed these steps:

1. Calculated allele frequencies: Determine the frequencies of each allele for the given gene in each group.
2. Calculated expected genotype frequencies: Using the allele frequencies, calculate the expected genotype frequencies assuming HWE.
3. Performed chi-square test: Compare the observed genotype frequencies with the expected frequencies using the chi-square test.
4. Interpreted results: Determine if the observed genotypes are significantly different from the expected genotypes, indicating deviations from HWE.

## 3. Results

### 3.1. Demographic Features

Age of the participant: The average age of the patient was 29.9 ± 2.3 years. Women with advanced maternal age are more likely to develop pre-eclampsia. “Advanced maternal age (AMA)” is defined as 35 years of age or older at the time of delivery. Meanwhile, “reproductive age (RA)” is considered less than 35 years. It was found that in group 1, 8 (24%) patients were in the AMA group whereas 25 (76%) were in the RA group, respectively. In Group 2, 6 (18%) patients were of AMA and 27 (82%) belonged to RA, while in Group 3, 10 (30%) participants were of AMA and 23 (70%) were of RA.

Onset of the disease: Considering the onset of the patient’s disease was also divided as having an early onset—developing symptoms before 34 weeks of gestation—or as having a late onset—developing symptoms after 34 weeks of gestation. In the present study, most of the pre-eclamptic subjects presented with early-onset disease where 23 (70%) patients were of early onset while 10 (30%) were of late onset in Group 1. In Group 2, 24 (73%) patients were of early onset and 9 (27%) were of late onset.

Body mass index: In the present study, most of the pregnant ladies had a higher BMI, which is one of the contributing factors in the development of the disease, and the same trend is also observed in pre-eclamptic women. A BMI of >30 kg/m^2^ was observed in 17 (52%) patients while <30 kg/m^2^ was observed in 16 (48%) in Group 1. In Group 2, a BMI of >30 kg/m^2^ was observed in 18 (55%) patients whereas <30 kg/m^2^ was observed in 15 (45%) patients. In Group 3, of the controls, a BMI of >30 kg/m^2^ was observed in 27 (82%) and <30 kg/m^2^ was observed in 06 (18%).

Gestational age at delivery: A gestational age of 36 weeks is considered safe in developing countries. The majority of the pre-eclamptic women presented after 36 weeks of gestation, although the range is between 25 and 38 weeks. In Group 1, five (15%) patients presented before 36 weeks and the rest presented after 36. In Group 2, seven (21%) presented before 36, and in Group 3, only one (3%) patient presented before 36 weeks.

Parity: Pre-eclampsia usually manifests in nulli/primipara (first-time pregnant) ladies as compared to multipara ladies. In this study, most of the pre-eclamptic patients presented as nulli/primipara. In Group 1, 22 (67%) pre-eclamptic patients were nulli/primipara and 11 (33%) were multipara. In Group 2, 27 (82%) were nulli/primiparous, whereas the rest of the 6 (18%) were multipara. In Group 3, of the controls, 7 pregnant ladies (21%) were nulli/primipara and 26 (79%) were multipara.

Socioeconomic status: Socioeconomically, most of the participants belonged to the middle class. In Group 1, 6 (18%) patients were from the poor class, 26 (79%) were from the middle, and only 1 (3%) was from the upper class. In Group 2, 7 (21%) belonged to the poor class and 26 (79%) belonged to the middle class. In Group 3, 7 (21%) were from the poor class and 26 (79%) were from the middle class socioeconomically. None of the patients presented from the upper class in Groups 2 and 3.

The demographic characteristics of the studied groups are illustrated in Table 2.

In the present study, an attempt has been made to analyze IL-6, IL-1β, and Apolipoprotein B-100 gene polymorphisms in pre-eclampsia and their association with the disease. For this, we selected 99 subjects for genetic studies, out of which 66 were cases and 33 were controls. The cases are further divided into two groups, with 33 in each based on the same grouping criteria, i.e., PE with severe hypertension and PE with hypertension. The blood samples were collected from selected study subjects. The genomic DNA was extracted (see Section 2). The results are presented under the following headings.

### 3.2. Purity of Chromosomal DNA from Selected Blood Samples

**Figure 1:**
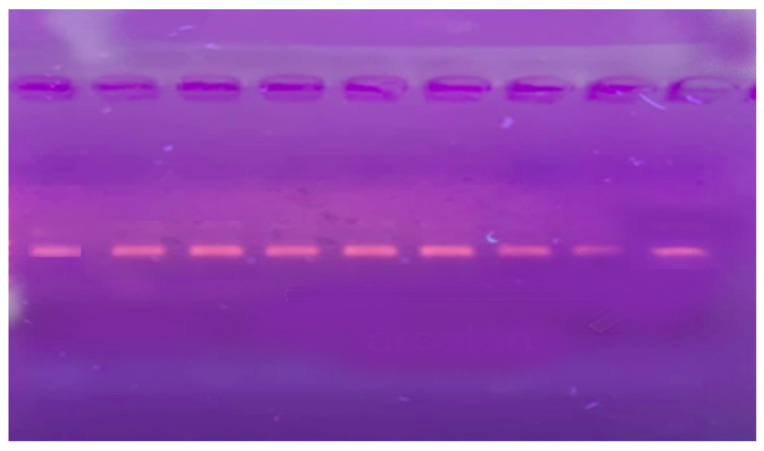
Extracted DNA on Agarose gel.

The electrophoretic analysis of the extracted chromosomal DNA from a blood sample is presented in Figure 1. The purity of genomic DNA isolated from the study samples was evaluated by electrophoretic analysis on 1% agarose gel. The visual observation revealed the presence of good-quality DNA in all the samples. However, the concentration of DNA varied in the samples. The result indicated that the DNA extraction method was good, and the extracted DNA was suitable for PCR amplification.

### 3.3. Polymerase Chain Reaction (PCR): Analysis of IL6, IL-1β, and Apolipoprotein B-100 Genes

**Figure 2:**
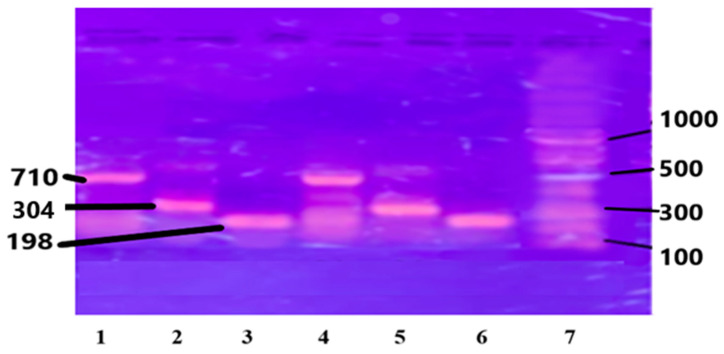
PCR amplified genes on Agarose gel: Lanes 1 and 4 are Apo B-100, 710 bp; Lanes 2 and 5 are IL-1β, 304 bp; Lanes 3 and 6 are IL-6, 198 bp; and Lane 7 is DNA ladder, 1000 bp.

The purified chromosomal DNA from the selected study samples was amplified using specific primers for genes IL-6, IL-1B, and Apo B-100 genes. (see Section 2). The results are presented in Figure 2.

### 3.4. Restriction Fragment Length Polymorphism (RFLP)

The purified and amplified product underwent digestion with specific restriction enzymes. In the case of the IL-6 gene, the SfaNI restriction enzyme was employed, resulting in the cleavage of the amplified DNA into two fragments measuring 140 and 58 base pairs (BPs), respectively. Similarly, for the IL-1β gene, the AvaI restriction enzyme was utilized, leading to the formation of two fragments sized at 114 and 190 BPs. For the Apo B-100 gene, digestion was performed using the XbaI enzyme, resulting in the generation of two fragments measuring 300 and 410 BPs.

**Figure 3:**
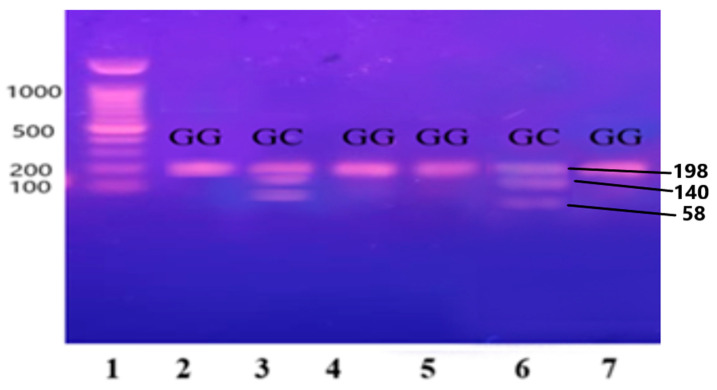
Gene IL-6 restricted with SfaNI restriction enzyme, resulting in the cleavage of the amplified DNA into two fragments measuring 140 and 58 base pairs (BPs) and separated on 12.5% non-denaturing polyacrylamide gel of pre-eclamptic patients.

**Figure 4:**
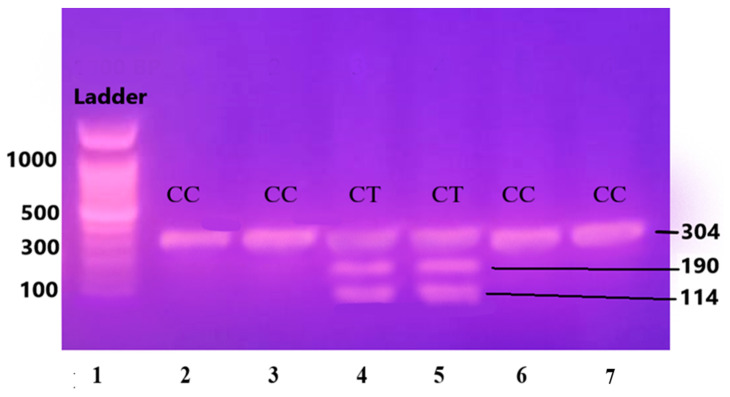
Gene IL-1β restricted with AvaI restriction enzyme leading to the formation of two fragments sized at 114 and 190 base pairs (BPs) and separated on 12.5% non-denaturing polyacrylamide gel of pre-eclamptic patients.

**Figure 5:**
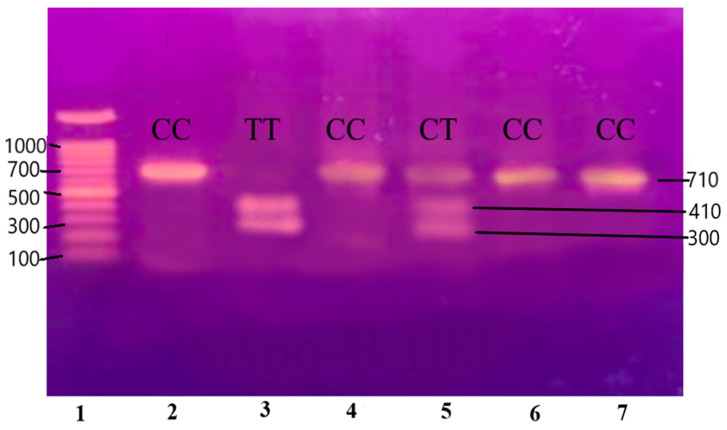
Gene Apo-B100 restricted with XbaI restriction enzyme resulting in the generation of two fragments measuring 300 and 410 base pairs (BPs) and separated on 12.5% non-denaturing polyacrylamide gel of pre-eclamptic patients.

The PCR-RFLP results are shown in Figure 3, Figure 4 and Figure 5.

Lane 1 1000 BP Ladder and Lanes 2, 4, 5, 7, 8, 9, and 11 indicate the presence of homozygous alleles (GG genotype). Lanes 3, 6, 10, and 12 show heterozygous alleles (GC genotype).

Lane 1 1000 BP Ladder and Lanes 2, 3, 6, and 7 show homozygous alleles (CC genotype). Lanes 4 and 5 show heterozygous alleles (CT genotype).

Lane 1 1000 BP Ladder and Lanes 2, 4, 5, 7, 8, 9, and 11 show homozygous alleles (GG genotype). Lanes 3, 6, 10, and 12 show heterozygous alleles (GC genotype).

### 3.5. Genetic Results/Allelic Frequencies

The results of restriction are employed in the form of allelic frequencies of the three genes IL-6 (-174G/C), IL-1β (-511 C/T), and Apo B-100 (2488 C/T), distributed in three groups, are shown in Table 3. The Hardy–Weinberg equilibrium with a chi-square test was applied to three, i.e., PE with severe HTN (Group 1), PE with HTN (Group 2), and control (Group 3) groups.

The allelic frequencies of the IL-6 genotypes CC, GC, and GG were 57.5%, 30.3%, and 12.2% in women with severe pre-eclampsia; 45.5%, 42.3%, and 12.2% in mild pre-eclamptic cases; and 33.3%, 57.5% and 9.2% in normal pregnant women, respectively. The allelic frequencies of IL-1β genotypes CC, CT, and TT were 66.6%, 30.3%, and 3.1% in severe pre-eclampsia patients; 60.6%, 36.4%, and 3.1% in mild cases; and 57.5%, 39.4% and 3.1% in normal pregnant women. The allelic frequencies of Apo B-100 genotypes CC, CT, and TT were 63.6%, 24.2%, and 12.1% in the severe disease group; 57.6%, 27.3%, and 15.1% in the mild disease group; and 56.0%, 32.0%, and 12.0% in the normal pregnant women.

Hardy–Weinberg equilibrium (HWE) analysis using chi-square tests indicated that IL6-174 G/C genotype frequencies deviated significantly from expected proportions in the combined analysis as shown in Table 4, possibly due to the population substructure. However, IL-1β-511 C/T and Apo B-100 2488 C/T genotypes were in HWE across all groups, suggesting a genetic equilibrium as shown in Table 5 and Table 6. The analysis indicated that the IL-6 gene is in disequilibrium according to the Hardy–Weinberg equation, which suggests an association of this gene with the severity of pre-eclampsia in the studied population.

## 4. Discussion

Pre-eclampsia is a complex disorder influenced by multiple factors in its pathogenesis and manifestations. This disease is characterized by endothelial dysfunction and a shift towards a persistent inflammatory response [3]. The extensive endothelial dysfunction and elevated systemic inflammatory response that define the maternal syndrome of pre-eclampsia are probably greatly influenced by elevated levels of pro-inflammatory cytokines, chemokines, and adhesion molecules in the mother’s bloodstream [33]. The production of these cytokines is regulated by their respective genes [22]. In this study, polymorphisms in the IL-6, IL-1β, and Apo B-100 genes were evaluated to investigate their potential association with the disease. Pregnant women with severe hypertension, hypertension, and no hypertension were enrolled for this purpose.

Starting with demographic and clinical characteristics in this study, several risk factors demonstrated notable differences and are reported. Most of the pre-eclamptic patients were older than the normotensive control subjects in the present study. Boghossian et al. (2014) previously reported that women over the age of 35 are at a higher risk of developing pre-eclampsia [34]. Additionally, Tyas et al. (2019) found a 4–5-fold increased risk of pre-eclampsia in women with advanced maternal age, which is associated with poor maternal and perinatal outcomes in pre-eclampsia patients [35]. In the present study, approximately 70% of patients reported early-onset pre-eclampsia, while around 30% had a late onset. Early-onset pre-eclampsia often involves a shallow trophoblast invasion in the spiral arteries, leading to syncytiotrophoblast stress, ischemic or reperfusion injuries, and inflammatory damage [7,8]. Similar findings were reported by You et al. (2018) in Taiwan [36]. The present study found that nulli/primiparity and multiparity occurred in 65% and 35% of Group 1, respectively, and in 81% and 19% of Group 2, respectively. This suggests that a significant number of women with pre-eclampsia were nulli/primiparous, indicating the disease is more prevalent in the first pregnancy. Similar findings were reported by Lisonkova et al. (2021) in the Canadian population [37]. In contrast, a study in Japan by Maeda et al. (2021) reported that multiparity was significantly associated with a lower incidence of pre-eclampsia [38]. In the current study, the proportion of overweight women was over 80% in the control group, whereas over half of the patients in both pre-eclamptic patient groups, i.e., 1 and 2, were overweight. According to a Canadian study by Wang et al. (2024), 33% of pregnancies were overweight and 67% of pregnancies had a normal BMI [38]. Nearly 80% of the patients in both pre-eclamptic groups delivered after 36 weeks of gestation, which is also consistent with the Canadian study [39]. The majority of subjects in all three analyzed groups exhibited a middle socioeconomic position, which is in line with a study by Lisonkova et al. (2021) [37].

Cytokines are thought to play a role in its pathogenesis [8,9,11]. Interleukin (IL) is one type of cytokine that alters biological responses in many diseases [40,41]. The study investigates the genetic underpinnings of pre-eclampsia, focusing on interleukin-6 (IL-6), interleukin-1 beta (IL-1β), and Apolipoprotein B-100 (Apo B-100) gene polymorphisms.

IL-6 is prominently expressed in the reproductive and gestational tissues of the female tract. It plays a varied role during pregnancy, influencing the development of the placenta and the necessary immunological adaptations for fetal tolerance [40]. Consequently, IL-6 is crucial in the pathophysiology of infertility and gestational disorders [41,42]. Elevated maternal blood levels of the cytokine IL-6 have been associated with preterm labour [22]. Women who have pre-eclampsia, on the other hand, have higher serum IL-6 levels and lower placental tissue IL-6 production [42,43]. While not necessary for a fruitful pregnancy, IL-6 is an important modulator of embryo implantation [43]. The present study reveals that IL6-174 G/C polymorphism shows a significant departure from the Hardy–Weinberg equilibrium (HWE) in pre-eclamptic patients, suggesting potential population admixture or specific patient characteristics influencing genotype frequencies. The same results were drawn by Puppala et al. (2016), where the -174G/C genotype in IL-6 has been linked to an increased risk of pre-eclampsia (PE) in the Indian population [44]. However, these findings contrast with numerous other studies that have assessed this SNP (single-nucleotide polymorphism) in Brazilian, Sri Lankan, Scottish, Turkish, Finish, Chinese, and American populations, where no association with the risk of pre-eclampsia (PE) was found [45,46,47,48,49,50,51]. IL-6 is a critical pro-inflammatory cytokine implicated in various disorders, including cardiovascular diseases and inflammation. Its genetic variant IL6-174 G/C has been linked to early-onset pre-eclampsia in previous studies, highlighting its role in genetic predisposition [52]. IL-6 is also associated with uric acid-induced inflammation in Gout [17]. A meta-analysis indicated the prognostic role of serum uric acid levels in pre-eclampsia [53]. Recently, serum uric acid-to-creatinine ratio (namely uric acid normalized for kidney function with the potential of selecting patients with hyperuricemia linked to ROS and inflammation) has been shown to associate with the development of PE [15]. The other pathway is the association between inflammatory cytokines and angiogenesis. A possible link between inflammation and angiogenesis is also represented by CD93, an angiogenic marker recently discovered in PE [54]. Angiogenic markers and balance are influenced by the genes and associated proteins analyzed in the present study.

IL1β-511 C/T and Apo B-100 2488 C/T genotypes did not significantly deviate from HWE across the studied groups. These findings align with those of another study by Pacheco-Romero conducted in Peru [30]. The IL-1β-511 C/T variant, involved in inflammatory pathways, and Apo B-100 2488 C/T, a marker in lipid metabolism, have been scrutinized for their associations with inflammatory diseases and metabolic disorders, respectively [20,55].

It is important to note that our findings should be validated in diverse populations with larger sample sizes to ensure their generalizability. This replication is crucial for understanding the pathogenesis of the disease. Future studies should also consider various demographic and genetic factors that may influence the incidence and progression of PE, thereby enhancing the precision of preventive measures.

Although the study has identified notable findings, it also has certain limitations such as being conducted in a single hospital; this may not represent the entire population of the country, potentially leading to ethnic bias. Additionally, quite a limited number of patients were included as the genetic studies are costly and there was no external funding.

## 5. Conclusions

Overall, while the IL-6-174 G/C variant shows an association with pre-eclampsia in our study, IL-1β-511 C/T and Apo B-100 2488 C/T do not exhibit significant associations. This underscores the complexity of genetic factors in pre-eclampsia and emphasizes the need for further research to elucidate their precise roles in disease pathogenesis and potential implications for personalized medicine approaches in clinical practice.

## Figures and Tables

**Table 1:** The Primer Set (Forward & Reverse) of Particular Gene for DNA Amplification.

No.	Gene	Primer Set	Product Size	Reference
1.	IL-6	F: 5′TGACTTCAGCTTTACTCTTTGT3′ R: 5′CTGATTGGAAACCTTATTAGG3′	198 bp	[30]
2.	IL-1β	F: 5TGGCATTGATCTGGTTCATC3′ R: 5’GTTTAGGAATCTTCCCACTT3’	304 bp	
3.	Apo B-100	F: 5’GGAGATATTCAGAAGCTAA3’ R: 5’GAAGAGCCTGAAGACTGACT3’	710 bp	

**Table 2:** Demographic characteristic of studied subjects in Group 1, 2 and 3.

No.	Characteristics	Group-1	Group-2	Group-3	*p*-Value
	No of Subjects	33	33	33	
1.	Age				<0.05
	>35 Years (AMA)	08 (24%)	06 (18%)	10 (30%)	
	<35 Years (RA)	25 (76%)	27 (82%)	23 (70%)	
2.	Onset of PE				<0.05
	Early Onset	23 (70%)	24 (73%)	Nil	
	Late Onset	10 (30%)	09 (27%)	Nil	
3.	BMI				<0.05
	>30 kg/m^2^	17 (52%)	18 (55%)	27 (82%)	
	<30 kg/m^2^	16 (48%)	15 (45%)	06 (18%)	
4.	Gestational Age at delivery				<0.05
	<36 weeks	05 (15%)	07 (21%)	01 (3%)	
	>36 weeks	28 (85%)	26 (79%)	32 (97%)	
5.	Parity				<0.05
	Nulli/primipara	22 (67%)	27 (82%)	07 (21%)	
	Multipara (>2)	11 (33%)	06 (18%)	26 (79%)	
6.	Socioeconomic status				<0.05
	Poor	06 (18%)	07 (21%)	07 (21%)	
	Middle	26 (79%)	26 (79%)	26 (79%)	
	Upper	01 (3%)	0 (0%)	0 (0%)	

**Table 3:** The allelic frequencies of the gene IL6-174 G/C, IL1B-511 C/T, and APOB100 2488 C/T polymorphism among the 3 groups.

Gene	Genotypes and Alleles	Group 1 *n* = 33	Group 2 *n* = 33	Group 3 *n* = 33	*p*-Value
IL6-174 G/C	CC	19 (57.5%)	15 (45.5%)	11 (33.3%)	<0.05
	CG	10 (30.3%)	14 (42.4%)	19 (57.5%)	
	GG	04(12.2%)	04 (12.2%)	3 (9.2%)	
	C	48 (72.7%)	44 (66.6%)	41 (62.1%)	
	G	18 (27.3%)	22 (33.3%)	25 (37.9%)	
IL1B-511 C/T	CC	22 (66.6%)	20 (60.6%)	19 (57.5%)	>0.05
	CT	10 (30.3%)	12 (36.4%)	13 (39.4%)	
	TT	1 (3.1%)	1 (3.1%)	1 (3.1%)	
	C	54 (81.8%)	52 (78.8%)	51 (77.3%)	
	T	16 (18.2%)	14 (21.2%)	15 (22.7%)	
APOB100 2488 C/T	CC	21 (63.6%)	19 (57.6%)	18 (56%)	>0.05
	CT	08 (24.2%)	09 (27.3%)	11 (32%)	
	TT	04 (12.1%)	05 (15.1%)	4 (12%)	
	C	50 (75.8%)	47 (71.2%)	47 (71.2%)	
	T	16 (24.2%)	19 (28.8%)	19 (28.8%)	

Analysis according to Hardy–Weinberg equilibrium.

**Table 4:** Hardy–Weinberg Analysis of IL6-174 G/C.

Group	Genotypes (CC/CG/GG)	Allele Frequencies (C/G)	Chi-Square (χ^2^\chi^2χ^2^)	HWE Interpretation
Group 1	19/10/4	0.727/0.273	1.95	In HWE (*p* > 0.05)
Group 2	15/14/4	0.666/0.333	0.083	In HWE (*p* > 0.05)
Group 3	11/19/3	0.621/0.379	1.697	In HWE (*p* > 0.05)
Overall	N/A	N/A	N/A	Deviates from HWE (*p* < 0.05)

**Table 5:** Hardy–Weinberg Analysis of IL1B-511 C/T.

Group	Genotypes (CC/CT/TT)	Allele Frequencies (C/T)	Chi-Square (χ^2^\chi^2χ^2^)	HWE Interpretation
Group 1	22/10/1	0.818/0.182	0.034	In HWE (*p* > 0.05)
Group 2	20/12/1	0.788/0.212	0.049	In HWE (*p* > 0.05)
Group 3	19/13/1	0.773/0.227	0.126	In HWE (*p* > 0.05)

**Table 6:** Hardy–Weinberg Analysis of APOB100 2488 C/T.

Group	Genotypes (CC/CT/TT)	Allele Frequencies (C/T)	Chi-Square (χ^2^\chi^2χ^2^)	HWE Interpretation
Group 1	21/8/4	0.758/0.242	3.622	In HWE (*p* > 0.05)
Group 2	19/9/5	0.712/0.288	3.575	In HWE (*p* > 0.05)
Group 3	18/11/4	0.712/0.288	1.050	In HWE (*p* > 0.05)

## Data Availability

Data supporting the reported results are available from the authors.
